# Objective acoustic voice outcomes following direct laryngochondroplasty in transgender women: a technique-specific cohort study

**DOI:** 10.1007/s00405-026-10567-4

**Published:** 2026-09-07

**Authors:** Eiman Abu Bandora, Michal Icht, Noa Diamant, Micalle Carl, Yotam Lior, Narin Nard Carmel Neiderman, Roni Vass, Hen Chaushu, Yuval Nachalon, Yael Oestreicher-Kedem

**Affiliations:** 1 Department of Otolaryngology Head and Neck Surgery and Maxillofacial Surgery, Tel-Aviv Sourasky University Medical Center, Tel Aviv, Israel; 2 Division of Anesthesiology and Critical Care, Tel-Aviv Sourasky University Medical Center, Tel Aviv, Israel; 3 Gray Faculty of Medical and Health Sciences, Tel Aviv University, Tel Aviv, Israel; 4 Department of Communication Disorders, Ariel University, Ariel, Israel

**Keywords:** Transgender, Women, Laryngochondroplasty, Voice, Outcome

## Abstract

**Purpose:**

To evaluate objective technique-specific voice outcomes following direct transvestibular laryngochondroplasty (DTVLCP) in transgender women.

**Methods:**

A retrospective cohort study conducted at a tertiary referral Center. Included were all transgender women who underwent DTVLCP from December 2019 to July 2025. Their pre- and postoperative voice recordings were analyzed using standardized acoustic parameters, including fundamental frequency (F0), formant frequencies (F1, F2), jitter, shimmer and maximum phonation time (MPT). Subgroup analyses compared patients with and without subjective voice changes and those undergoing isolated versus combined procedures.

**Results:**

Voice recordings of 28 transgender women (mean age 27.8 years) were available for acoustic analysis. Postoperative vocal fold movement was preserved in all patients. Seven patients reported subjective voice change. A comparison of pre- and postoperative acoustic analysis of the cohort’s voice recordings revealed no statistically significant changes in the measured acoustic parameters (mean F0, F1, F2, jitter, shimmer, and maximum phonation time). No significant acoustic changes were found between the subgroup of patients who reported subjective postoperative voice changes (*n* = 7) and that of patients who did not (*n* = 21), and between patients who underwent isolated DTVLCP (*n* = 9) and those who had the procedure combined with lower facial feminization surgery (*n* = 19).

**Conclusion:**

DTVLCP was not associated with significant objective changes in the measured acoustic voice parameters. These findings support the safety of DTVLCP with respect to voice and provide technique-specific evidence in a field where existing data are largely derived from subjective reports and heterogeneous surgical approaches.

## Introduction

Laryngochondroplasty (LCP) is a gender-affirming surgical procedure aiming to reduce the prominence of the thyroid cartilage. As feminizing hormone therapy does not induce structural remodeling of post-pubertal laryngeal cartilage, surgery remains the primary option for achieving a feminized neck contour in transgender women [1, 2]. LCP was originally described by Wolfort et al. using a transcervical approach [3]. Subsequent technical modifications sought to minimize cervical scarring. In 2020, the endoscopic transvestibular approach, initially developed for thyroidectomy, was adapted for LCP [4–6]. More recently, a direct transvestibular (DTVLCP) approach was introduced, allowing direct visualization and reduction of the thyroid cartilage through a subdermal tunnel from the oral vestibule, thereby eliminating the need for endoscopy [7, 8]. Using a drill, the anterior thyroid notch and superior thyroid cartilage margins are reduced under direct visualization. Care is taken to avoid reduction below a point 2 mm above the mid-height of the thyroid cartilage [9] and to preserve the integrity of the inner thyroid cartilage cortex anteriorly, thereby minimizing the risk of disrupting the anterior commissure attachment. The extent of cartilage resection is determined by intraoperative judgment, aimed at achieving an appropriate aesthetic reduction while maintaining laryngeal structural stability.

Voice constitutes a critical gender cue and a central component of gender identity in transgender women. Vocal incongruence has been associated with significant psychosocial distress and may adversely affect quality of life [10–12]. Although LCP is increasingly performed as part of gender-affirming care, its impact on vocal outcomes remains incompletely characterized. Existing studies report voice changes infrequently and predominantly rely on subjective assessments [13–18], with objective acoustic analyses reported in only a few publications [19–21]. Objective acoustic data are limited and inconsistent, with one series including 15 patients undergoing transcervical LCP, another reporting a single case, and a third describing a heterogeneous cohort 35 LCP procedures. Notably, however, only 4 cases in the latter involved DTVLCP, underscoring the paucity of DTVLCP-specific data. Building upon our initial description of the DTVLCP technique and subsequent clinical series, and given the central role of voice in gender dysphoria, the theoretical risk of phonatory alteration following LCP, and the paucity of objective data, particularly following newer LCP surgical techniques, this study aims to report technique-specific objective voice outcomes in a homogeneous cohort of transgender women undergoing DTVLCP.

## Methods

### Study design and ethics

A retrospective cohort study was conducted at the Tel Aviv Sourasky University Medical Center (TASUMC), a tertiary referral center. The study was approved by the institutional Helsinki committee (TLV-046-22), which waived informed consent for this retrospective anonymized analysis.

### Study population

Included were all consecutive self-identifying adult (≥ 18 years, *n* = 33) transgender women who underwent DTVLCP at a tertiary medical center between December 2019 and July 2025. The surgical technique has been previously described by our group [7]. Excluded from the final analysis were 5 patients: three who did not return to postoperative follow-up and 2 with missing preoperative voice recordings.

### Study procedures

The medical records of all study participants were reviewed and data on their demographics, surgical history, operative and postoperative courses, pre- and postoperative subjective patient self-reported voice symptoms and vocal fold mobility observed on flexible laryngoscopy were retrieved. Their pre- and postoperative voice recordings were acoustically analyzed with the Praat software version 6.2.01 (see supplement 1 for the voice recording protocol). The following voice indices were extracted: fundamental frequency (F0), F1, F2, jitter, and shimmer of the vowels /i/ and /a/, F0 of a 1 to 10 counting assignment and the F0 of the first sentence of the phonetically balanced Hebrew reading passage “Thousand Islands” [22]. The maximum phonation time (MPT) of three trials of /i/ was calculated.

### Outcome measures

The characteristics of the study population and their subjective voice reports were extracted from the medical records. Vocal fold mobility was determined based on information available in the medical files, and pre- and postoperative F0, F1, F2, jitter, shimmer, and MPT values were compared.

### Statistical analyses

The data collected for this study were recorded as summary tables. The statistical analysis was performed using Microsoft Excel version 16. Continuous variables were presented as mean, and standard deviation (SD) or median. Descriptive statistics were reported as percentages. The findings of pre- and postoperative acoustic analyses were compared using the Wilcoxon signed-rank test and reported as mean, SD and median. The Mann-Whitney U test was used for subgroups (isolated vs. combined DTVLCP and subjective voice change vs. no subjective voice change) comparisons and performed on delta-change (post-to-preoperative) and reported as median and interquartile range (IQR). Spearman’s rank correlation was used to assess the correlation between the post-to-preoperative change in acoustic scores and the postoperative recording interval (as a continuous variable) and reported as Spearman’s rho. An α of *P* < .05 was used to define statistical significance. To account for multiple comparisons across the 13 acoustic outcome measures, while minimizing excessive inflation of Type II error, *P*-values were adjusted using the Benjamini–Hochberg false discovery rate (FDR) procedure, with an FDR threshold of 0.05. All *P*-values were rounded to three decimal places.

## Results

### Study population

Twenty-eight patients (mean age 27.8 years, range 19–47 years) were included in the study. Nineteen DTVLCPs were performed in combination with lower facial feminization surgery, and nine were isolated procedures. One case was a revision after an endoscopic LCP. All study patients presented pre- and postoperative intact vocal fold motion as observed by flexible fiberoptic laryngoscopy.

### Self-reported voice outcome

Seven patients reported postoperative voice changes, including a reduced vocal range (*n* = 3), voice fatigue (*n* = 1), and one each of a more feminine, more masculine, and more throaty voice. Within this group of patients, postoperative follow-up by flexible fiberoptic laryngoscopy did not reveal any laryngeal anatomical abnormalities.

### Acoustic analysis

The results of the pre- and postoperative acoustic analyses are presented in Table 1. Preoperatively, the median F0 for /i/ /a/ (182.76 Hz and 168.32 Hz, respectively) were higher than expected in cis males. The postoperative values of F0 of /a/ significantly increased (159.50 Hz vs. 183.53 Hz, *P*=.01), however, this finding did not remain significant following FDR correction.

**Table 1:** Comparison of pre- and postoperative acoustic parameters using Wilcoxon signed-rank tests for paired comparisons

Parameter	Pre Mean ± SD	Pre Median	Post Mean ± SD	Post Median	*P*-value	FDR adjusted *P*-value
F0 (/i/) Hz	182.76 ± 52.87	168.92	188.45 ± 42.90	186.28	0.17	0.55
F1 (/i/) Hz	331.25 ± 40.45	332.15	332.25 ± 49.36	326.53	0.95	0.95
F2 (/i/) Hz	2407.48 ± 199.71	2394.19	2374.69 ± 222.32	2362.42	0.80	0.95
Jitter (/i/) %	0.36 ± 0.19	0.31	0.296 ± 0.13	0.27	0.20	0.55
Shimmer (/i/) %	1.87 ± 1.00	1.59	1.96 ± 0.97	1.7	0.64	0.95
F0 (/a/) Hz	168.32 ± 39.64	159.50	184.02 ± 38.41	183.53	0.01	0.13
F1 (/a/) Hz	729.00 ± 146.65	744.60	723.95 ± 130.60	741.06	0.92	0.95
F2 (/a/) Hz	1453.87 ± 106.63	1468.23	1453.88 ± 138.42	1485.22	0.70	0.95
Jitter (/a/) %	0.338 ± 0.180	0.31	0.3 ± 0.12	0.26	0.35	0.57
Shimmer (/a/) %	2.26 ± 1.05	2.17	1.99 ± 1.121	1.71	0.33	0.57
Counting F0 Hz	153.11 ± 32.07	147.67	153.17 ± 30.32	147.81	0.79	0.95
Reading F0 Hz	146.03 ± 26.82	138.46	147.43 ± 28.42	141.49	0.25	0.55
MPT (sec)	14.75 ± 6.28	13.31	15.40 ± 5.23	15.16	0.25	0.55

*F* Fundamental frequency, *MPT* maximal phonation time, *SD* standard deviation, *FDR* Benjamini–Hochberg false discovery rate

There were no statistically significant differences in the post-to-preoperative acoustic parameters between the group of patients who reported subjective postoperative voice change (*n* = 7) and the group of patients who did not (*n* = 21), and between the group of patients who underwent combined DTVLCP (*n* = 19) and the group of isolated DTVLCP (*n* = 9) (Tables 2 and 3).

**Table 2:** Subgroup comparison of the median delta acoustic change according to subjective voice assessment. *P*-values are derived from Mann-Whitney U tests

Parameter	No subjective voice change (*n* = 21) Median Delta (IQR)	Subjective voice change (*n* = 7) Median Delta (IQR)	*P*- value
F0 (/i/), Hz	12.84 (-3.44-18.43)	1.47 (-12.14-11.53)	0.27
F1 (/i/), Hz	-0.03 (-29.38-42.74)	-15.50 (-41.80-34.59)	0.44
F2 (/i/), Hz	36.94 (-51.88-102.11)	-33.30 (-80.67-43.11)	0.19
Jitter (/i/), %	-0.08 (-0.24-0.08)	0.03 (-0.16-0.06)	0.89
Shimmer (/i/), %	0.01 (-0.88-0.93)	-0.13 (-0.38-0.96)	0.76
F0 (/a/), Hz	10.97 (3.24–40.12)	3.88 (-7.47- 22.01)	0.21
F1 (/a/), Hz	19.43 (-61.24-103.59)	-41.21 (-87.16- [-4.60])	0.27
F2 (/a/), Hz	-4.69 (-72.75-56.42)	66.48 (-71.24-138.60)	0.30
Jitter (/a/), %	-0.0045 (-0.07-0.09)	-0.042 (-0.18-0.0025)	0.18
Shimmer (/a/), %	-0.22 (-1.18-0.43)	-0.45 (-1.39-1.39)	0.60
Counting F0, Hz	2.03 (-5.97-6.40)	4.65 (-20.80-8.07)	0.68
Reading F0, Hz	1.42 (-3.88-8.04)	7.05 (-2.49-9.94)	0.60
MPT, sec	1.21 (-1.21-4.07)	-0.596 (-0.90-2.08)	0.44

*F* Fundamental frequency, *MPT* maximal phonation time, *IQR* interquartile range

**Table 3:** Subgroup comparison of the median delta acoustic change according to surgery type: isolated DTVLCP versus combined with other facial feminization procedures. *P*-values are derived from Mann-Whitney U tests

Parameter	Isolated (*n* = 9) Median Delta (IQR)	Combined (*n* = 19) Median Delta (IQR)	*P*- value
F0 (/i/), Hz	-0.63 (-12.14-12.02)	12.84 (-0.79-23.05)	0.11
F1 (/i/), Hz	-3.94 (-45.73-16.7)	-2.38 (-17.04-42.74)	0.41
F2 (/i/), Hz	26.71 (-99.67-44.57)	25.70 (-37.20-79.44)	0.47
Jitter (/i/), %	0.06 (-0.10-0.61)	-0.81 (-0.24-0.10)	0.81
Shimmer (/i/), %	0.06 (-0.37-0.90)	-0.13 (-0.73-0.94)	0.81
F0 (/a/), Hz	8.30 (-7.47-18.01)	9.59 (3.24–35.67)	0.47
F1 (/a/), Hz	-83.97 (-169.00-43.61)	13.15 (-36.94-103.59)	0.11
F2 (/a/), Hz	20.08 (-105.96-66.48)	-4.69 (-50.82-86.62)	0.96
Jitter (/a/), %	-0.0045 (-0.07-0.04)	-0.005 (-0.10-0.04)	1
Shimmer (/a/), %	-0.39 (-1.05-0.85)	-0.22 (-1.18-0.40)	0.66
Counting F0, Hz	2.03 (-7.18-6.66)	4.25 (-5.07-9.23)	0.68
Reading F0, Hz	3.55 (-3.34-9.21)	0.62 (-2.78-1.73)	0.60
MPT, sec	2.08 (-1.07-4.39)	0.89 (-0.78-2.89)	0.92

*F* Fundamental frequency, *MPT* maximal phonation time, *IQR* interquartile range

Postoperative voice recordings were obtained at a mean of 4.8 months (range 0.5–66 months, SD 11.6 months, median 1.7 months). Seventeen patients were recorded within 2 months postoperatively, 8 between 2 and 5 months, and 3 between 8 and 66 months postoperatively. Spearman’s correlation analysis demonstrated a moderate positive correlation between postoperative recording interval and the post-to-preoperative change in F2 of /a/ (*ρ*=0.554, *P*=.002), indicating a greater increase in F2 /a/ values at longer follow-up intervals (Table 4).

**Table 4:** Spearman correlation between postoperative recording interval and post-to-preoperative acoustic change scores

Parameter (Δ Post–Pre)	Spearman’s ρ	*P*-value	FDR adjusted *P*-value
F0 (/i/) Hz	0.26	0.17	0.55
F1 (/i/) Hz	0.25	0.21	0.55
F2 (/i/) Hz	0.01	0.95	0.95
Jitter (/i/) %	-0.10	0.63	0.95
Shimmer (/i/) %	0.16	0.41	0.57
F0 (/a/) Hz	0.05	0.79	0.94
F1 (/a/) Hz	-0.04	0.85	0.95
F2 (/a/) Hz	0.55	0.002*	0.026*
Jitter (/a/) %	-0.20	0.31	0.55
Shimmer (/a/) %	0.36	0.06	0.39
Counting F0 Hz	0.05	0.79	0.15
Reading F0 Hz	0.22	0.26	0.94
MPT (sec)	-0.43	0.02	0.55

*F* Fundamental frequency, *MPT* maximal phonation time, *FDR* Benjamini–Hochberg false discovery rate

*Statistically significant

## Discussion

Building on our initial description of the DTVLCP, this study reports technique-specific objective acoustic voice outcomes in a homogeneous cohort. While LCP gains broader clinical adoption, including direct transvestibular and endoscopic techniques, quantitative evaluation of vocal outcomes following LCP remains scarce, with most published work relying on subjective assessments and mixed cohorts [8, 13–18, 21, 23–28]. Given the central role of voice in gender identity among transgender individuals and the potential risk of voice alterations following LCP, objective characterization of the procedure’s impact on voice is essential, specifically following DTVLCP, an increasingly utilized approach.

In our cohort, no statistically significant changes were observed in 12 of the 13 acoustic parameters assessed. Postoperatively, an increase in F0 of /a/ phonation was observed; however, this increase did not remain statistically significant following FDR correction (adjusted *P*=.13). Several mechanisms may theoretically account for the observed nominal increase in F0 during sustained /a/ phonation. Although the increase did not remain significant after FDR correction, several speculative mechanisms could potentially account for such a finding. Reduction of the anterior thyroid cartilage prominence could decrease the effective mass of the laryngeal framework, contributing to F0 elevation despite the cartilage not forming part of the vibratory vocal fold unit [29]. Alternatively, postoperative scar formation along the transvestibular access tunnel may result in subtle anterior displacement or increased tension within the laryngeal framework. Behavioral or phonatory adaptation following surgery may also play a role. However, these mechanisms remain speculative, and the lack of consistent F0 changes across vowels and speech tasks warrants caution.

In contrast to the slight increase observed in F0 of /a/ phonation, F0 remained unchanged for all other tasks. Also important, jitter and shimmer, widely accepted indicators of phonatory stability and vibratory regularity, showed no significant postoperative changes for either the /a/ or /i/ vowels, suggesting preserved vocal fold vibratory function.

Overall, our findings indicate that LCP did not result in significant objective acoustic changes. As DTVLCP does not directly involve manipulation of the vocal folds or cricothyroid mechanism, the absence of significant post-to-preoperative differences across all acoustic parameters supports the premise that anterior thyroid cartilage contouring can be performed without a detrimental effect on phonatory control. These findings align with previous, predominantly subjective, reports [6–8, 13–19, 23–25] and are further corroborated by the absence of structural alterations to the vocal folds or disruption of the anterior commissure observed intraoperatively. Larger cohorts with longitudinal follow-up are needed to determine whether our results represent reproducible effects.

Another observation in our study was the elevated preoperative mean F0 values for /i/ and /a/ (182.76 Hz and 168.32 Hz, respectively) than expected for cis males. The F0 of voice is primarily determined by the length, tension, and mass of the vocal folds [30]. In general, males have longer and thicker vocal folds, which vibrate more slowly and produce a lower F0 [31–33]. The average speaking F0 of adult males reportedly ranges from approximately 107 to 120 Hz. Females, on the other hand, have shorter and thinner vocal folds that vibrate more rapidly, resulting in a higher F0, with an average F0 between 189 and 224 Hz [34–36]. For biological males to be perceived as females, their F0 must reportedly increase to at least 155 to 165 Hz, and potentially as high as 180 Hz to be considered “feminine” [37–39]. Similar to our observation, the relatively high preoperative F0 has also been reported by Aries et al., with an average of 171.3 HZ in a cohort of 11 patients undergoing transcervical LCP [19]. Several factors may contribute to the F0 of transgender women being higher than expected for that of cisgender men [32]. Transgender women often undergo speech therapy and voice training to modify their vocal pitch and resonance. There are various techniques by which they learn to speak at a higher pitch and adjust their speaking patterns to align more closely with typical female speech [40]. Also, to conform to social gender norms, transgender women may consciously or unconsciously adopt a higher pitch when speaking.

Seven patients reported subjective voice changes following LCP. However, these changes were not accompanied by any objective acoustic changes or by laryngoscopy findings. These subjective outcomes highlight the complexity of voice perception and suggest that subjective voice experiences may not be fully captured by the acoustic measures examined in the present study, as well as the need for individualized patient counseling and management of expectations before surgery. Also, the extent of surgery (isolated DTVLCP vs. DTVLCP combined with lower facial feminization surgery) did not influence voice outcomes.

Due to the retrospective nature of the study design and variable adherence to postoperative follow-up, the timing of postoperative voice recording intervals was not uniform. Although most patients (*n* = 17, 60.7%) were recorded within 2 months postoperatively, the remaining patients were recorded at later time points. To evaluate the potential impact of time from surgery on acoustic outcomes, the correlation between postoperative recording interval and acoustic change scores was examined. A moderate positive correlation was observed in F2 of /a/. This finding should be interpreted cautiously given the exploratory nature of the analysis and the small number of participants with longer follow-up. It may reflect time-dependent postoperative changes in vocal tract configuration or articulatory-resonance adaptation rather than a direct effect on phonatory frequency. However, as laryngeal position and vocal tract morphology were not directly assessed, the mechanism underlying this finding cannot be determined in the present study.

There are several limitations to this study. It involved a single institution cohort, and the vocal results may not apply to other LCP surgical techniques. There is also variability in the period from DTVLCP to the postoperative voice recording, as well as in the surgical procedures (isolated or combined DTVLCP). Additionally, the short follow-up period for some patients may not capture long-term voice outcomes. Although subjective and perceptual voice measures were not included, the use of objective acoustic parameters provides a focused evaluation of phonatory function, reducing the influence of perceptual and psychosocial factors. Future studies should aim for larger cohorts, consistent timing of postoperative voice recordings and longer follow-up periods to better understand the durability of voice changes post-DTVLCP.

## Conclusion

This is the largest study to report objective DTVLCP technique-specific acoustic voice outcomes. No objective deterioration in the measured acoustic voice parameters was observed. These findings provide clinically relevant objective evidence supporting the safety profile of DTVLCP and to its role as a reliable technique in gender-affirming laryngeal surgery.

## Data Availability

The data that support the findings of this study are not publicly available due to ethical restrictions but are available from the corresponding author on reasonable request and subject to institutional approval.

## References

[1] Nuyen BA, Qian ZJ, Campbell RD, Erickson-DiRenzo E, Thomas J, Sung CK (2022) Feminization laryngoplasty: 17‐year review on long‐term outcomes, safety, and technique. Otolaryngol Head Neck Surg 167(1):112–117. 10.1177/01945998211036870 34399638

[2] Van Rossem AP, Meijer BA, Rinkel RNPM (2021) Recommended maximum laryngeal prominence size in adult females: A cross-sectional study proposing a laryngeal prominence size standard for chondrolaryngoplasty in male-to-female transgender individuals. Plast Reconstr Surg 147(4):935–945. 10.1097/PRS.0000000000007785 33761516

[3] Wolfort FG, Parry RG (1975) Laryngeal chondroplasty for appearance. Plast Reconstr Surg 56(4):371–374. 10.1097/00006534-197510000-00000 1161910

[4] Chung J, Purnell P, Anderson S, Hoelke C, Bender-Heine A, Lambert HW (2020) Transoral chondrolaryngoplasty: scarless reduction of the adam’s apple. OTO Open 4(3):2473974X20938299. 10.1177/2473974X20938299/7 PMC7359653 32704609

[5] Verhasselt M, Cavelier G, Horoi M, Dequanter D, Rodriguez A (2020) Chondrolaryngoplasty for transgender patients: feasibility of a scar-free approach. Eur Arch Otorhinolaryngol 277(8):2381–2384. 10.1007/s00405-020-06020-9 32385735

[6] Khafif A, Shoffel-Havakuk H, Yaish I, Tordjman K, Assadi N (2020) Scarless neck feminization: Transoral transvestibular approach chondrolaryngoplasty. Facial Plast Surg Aesthet Med 22(3):172–180. 10.1089/fpsam.2020.0021 32101468

[7] Neiderman NNC, Arbel S, Diamant N, Wolfman K, Yanko R, Lior Y, Kidron A, Ianculovici C, Kleinman S, Oestreicher-Kedem Y (2024) Direct trans-vestibular feminization thyroid laryngochondroplasty. Plast Reconstr Surg 153(2):467–476. 10.1097/PRS.0000000000010560 37075278

[8] David AP, Knott PD, Rosen CA, Young VN, Park AM, Seth R (2022) Clinical feasibility and efficacy of the externally scarless transoral chondrolaryngoplasty. Facial Plast Surg Aesthet Med 24(S2):S41–S43. 10.1089/fpsam.2021.0295 35506892

[9] Jahnavi, Thomas JP (2026) Anterior commissure position relative to the thyroid cartilage for safe chondrolaryngoplasty. Laryngoscope 136(5):2278–2283. 10.1002/lary.70325 41450226

[10] Bachorowski J-A, Owren MJ (1999) Acoustic correlates of talker sex and individual talker identity are present in a short vowel segment produced in running speech. J Acoust Soc Am 106(2):1054–1063. 10.1121/1.427115 10462810

[11] Knudson G, De Cuypere G, Bockting W (2010) Recommendations for revision of the DSM diagnoses of gender identity disorders: Consensus statement of the World Professional Association for Transgender Health. Int J Transgend 12(2):115–118. 10.1080/15532739.2010.509215

[12] Scherer KR (1986) Vocal affect expression: A review and a model for future research. Psychol Bull 99(2):143–165. 10.1037/0033-2909.99.2.143 3515381

[13] Conrad K, Yoskovitch A (2003) Endoscopically facilitated reduction laryngochondroplasty. Arch Facial Plast Surg 5(4):345–348. 10.1001/archfaci.5.4.345 12873874

[14] Al-Jassim AHH, Lesser THJ (2006) Reduction of Adam’s apple for appearance. Indian J Otolaryngol Head Neck Surg 58(2):172–173. 10.1007/BF03050778 PMC3450779 23120275

[15] Spiegel JH, Rodriguez G (2008) Chondrolaryngoplasty under general anesthesia using a flexible fiberoptic laryngoscope and laryngeal mask airway. Arch Otolaryngol Head Neck Surg 134(7):704–708. 10.1001/archotol.134.7.704 18645118

[16] Cohen MB, Insalaco LF, Tonn CR, Spiegel JH (2018) Patient satisfaction after aesthetic chondrolaryngoplasty. Plast Reconstr Surg Glob Open 6(10):e1877. 10.1097/GOX.0000000000001877 PMC6250475 30534483

[17] Tang CG (2020) Evaluating patient benefit from laryngochondroplasty. Laryngoscope 130(S5):S91. 10.1002/lary.29075 32978785

[18] Strickland L, Sussman KA, Madden LL (2025) Vocal quality complication following chondrolaryngoplasty: A reported case. J Voice 39(3):856e9–856e11. 10.1016/j.jvoice.2022.11.011 36456356

[19] Aires MM, de Vasconcelos D, de Moraes BT (2020) Chondrolaryngoplasty in transgender women: Prospective analysis of voice and aesthetic satisfaction. Int J Transgend Health 22(4):394–402. 10.1080/26895269.2020.1848690 PMC10553374 37808531

[20] Hughes C, Brown G, Ma N, Dibben N (2024) Acoustic effects of facial feminisation surgery on speech and singing: a case study. Interspeech. ; September 1–5 2024; Kos island, Greece. Available from: https://www.isca-archive.org/interspeech_2024/hughes24_interspeech.pdf. Accessed May 5, 2025

[21] Dwyer CD, Fein M, Kridgen S, Winston J (2026) Chondrolaryngoplasty: vocal considerations, outcomes, and effects on acoustic measures of voice. Laryngoscope 1–8. 10.1002/lary.70557 PMC13460668 41963097

[22] Amir O (2016) Thousand Islands – Hebrew reading passage: preparation and validation. 10.13140/RG.2.1.2837.0809

[23] Eggerstedt M, Lee JC, Mendelsohn AH (2024) Transoral feminizing chondrolaryngoplasty: Development and deployment of a novel approach in 77 patients. Facial Plast Surg Aesthet Med 26(5):618–625. 10.1089/fpsam.2022.0016 35704281

[24] Nuyen B, Qian ZJ, Rakkar M, Thomas JP, Erickson-DiRenzo E, Sung CK (2023) Diagnosis and management of vocal complications after chondrolaryngoplasty. Laryngoscope 133(9):2301–2307. 10.1002/lary.30518. Epub 2022 Dec 28 36576093

[25] Shoffel-Havakuk H, Cohen O, Lahav Y, Khafif A (2023) Scarless neck feminization by transoral endoscopic vestibular approach chondrolaryngoplasty: A prospective cohort. Otolaryngol Head Neck Surg 169(1):31–40. 10.1002/ohn.296. Epub 2023 Feb 17 36802074

[26] Zeng Y, Jiao H, Yang J et al (2023) A new technique for chondrolaryngoplasty in patients assigned female at birth. Ann Plast Surg 90(1):56–60. 10.1097/SAP.0000000000003324 36534101

[27] Kondamuri NS, Mallur PS, Courey MS, Naunheim MR (2023) Complications and functional voice limitations following gender-affirming laryngeal surgery (GALS). J Voice 11:S0892 1997(23)00291-6 10.1016/j.jvoice.2023.09.015 37957071

[28] Abu Bandora E, Chaushu H, Icht M, Carmel Neiderman NN, Lior Y, Vass R, Nachalon Y, Oestreicher-Kedem Y (2026) Transgender women voice outcome after laryngochondroplasty - a systematic literature review. Oto Open 10:e70264. 10.1002/oto2.70264 PMC13313092 42376589

[29] Arfken GB, Griffing DF, Kelly DC, Priest J (1984) University Physics (Mechanical waves). Elsevier Sci 344–360. 10.1016/B978-0-12-059860-1.50023-0

[30] Riede T, Brown C (2013) Body size, vocal fold length, and fundamental frequency – implications for mammal vocal communication. Nova Acta Leopoldina NF 111:295–314

[31] Calvert DR (1988) Clinical measurement of speech and voice, by Ronald J. Baken, PhD, 528 pp, paper, College-Hill Press, Boston, MA, 1987, $35.00. Laryngoscope 98:905–906. 10.1288/00005537-198808000-00028

[32] Hunter EJ, Tanner K, Smith ME (2011) Gender differences affecting vocal health of women in vocally demanding careers. Logoped Phoniatr Vocol 36(3):128–136. 10.3109/14015439.2011.587447 PMC3306615 21722077

[33] Titze IR (1987) Physiology of the female larynx. J Acoust Soc Am 82(S1):S91. 10.1121/1.2025049

[34] Hancock AB, Garabedian LM (2013) Transgender voice and communication treatment: A retrospective chart review of 25 cases. Int J Lang Commun Disord 48(1):54–65 10.1111/j.1460-6984.2012.00185.x 23317384

[35] Adessa M, Weston Z, Ruthberg J, Bryson PC (2023) Gender-affirming voice modification for transgender women: characteristics and outcomes. Transgender Health 8(4):348–356. 10.1089/trgh.2021.0071 PMC10387149 37525833

[36] Colton RH, Casper JK, Leonard R (2006) Understanding voice problems: A physiological perspective for diagnosis and treatment, Third edn. Lippincott Williams & Wilkins, Baltimore, Maryland, pp 22–23

[37] Wolfe VI, Ratusnik DL, Smith FH, Northrop G (1990) Intonation and fundamental frequency in male-to-female transsexuals. J Speech Hear Disord 55(1):43–50. 10.1044/jshd.5501.43 2299839

[38] Gelfer MP, Schofield KJ (2000) Comparison of acoustic and perceptual measures of voice in male-to-female transsexuals perceived as female versus those perceived as male. J Voice 14(1):22–33. 10.1016/S0892-1997(00)80092-2 10764114

[39] Gorham-Rowan M, Morris R (2006) Aerodynamic analysis of male-to-female transgender voice. J Voice 20(2):251–262. 10.1016/j.jvoice.2005.03.004 16112545

[40] Adler RK, Hirsch S, Pickering J (eds) (2018) Voice and Communication Therapy for the Transgender/Gender Diverse Client: A comprehensive clinical guide. Plural Publishing, San Diego, CA

